# From Nutritional Patterns to Behavior: High-Fat Diet Influences on Inhibitory Control, Brain Gene Expression, and Metabolomics in Rats

**DOI:** 10.1021/acschemneuro.4c00297

**Published:** 2024-11-28

**Authors:** Diego Ruiz-Sobremazas, Ana Cristina Abreu, Ángeles Prados-Pardo, Elena Martín-González, Ana Isabel Tristán, Ignacio Fernández, Margarita Moreno, Santiago Mora

**Affiliations:** † Center for Welfare and Social Inclusion of the University of Almeria, Crta. Sacramento s/n, La Cañada de San Urbano 04120, Spain; ‡ Department of Psychology and Sociology, University of Zaragoza, Crta. Atarazana 4, Teruel 44003, Spain; § Department of Chemistry and Physics, Research Center CIAIMBITAL, University of Almería, Crta. Sacramento s/n, La Cañada de San Urbano 04120, Spain; ∥ Current: School of Psychology and Neuroscience, University of St. Andrews, St Mary’s Quad, South St., St Andrews KY16 9JP, United Kingdom

**Keywords:** high-fat diet, impulsivity, metabolomics, decision-making, inhibitory control, brain gene expression

## Abstract

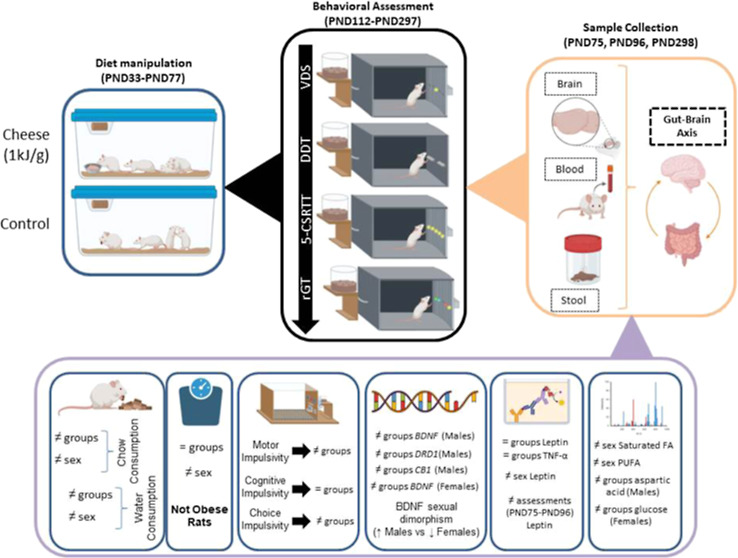

Impulsive and compulsive behaviors are associated with inhibitory control deficits. Diet plays a pivotal role in normal development, impacting both physiology and behavior. However, the specific effects of a high-fat diet (HFD) on inhibitory control have not received adequate attention. This study aimed to explore how exposure to a HFD from postnatal day (PND) 33 to PND77 affects impulsive and compulsive behaviors. The experiment involved 40 Wistar rats subjected to HFD or chow diets. Several tasks were employed to assess behavior, including variable delay to signal (VDS), five choice serial reaction time task (5-CSRTT), delay discounting task (DDT), and rodent gambling task (rGT). Genetic analyses were performed on the frontal cortex, and metabolomics and fatty acid profiles were examined by using stool samples collected on PND298. Our results showed that the HFD group exhibited increased motor impulsive behaviors while not affecting cognitive impulsivity. Surprisingly, reduced impulsive decision-making was shown in the HFD group. Furthermore, abnormal brain plasticity and dopamine gene regulation were shown in the frontal cortex, while metabolomics revealed abnormal fatty acid levels.

## 1 Introduction

Inhibitory control is defined as the ability to inhibit or control impulsive and/or compulsive responses.^1^ Impulsivity and compulsivity are cardinal clinical features of several neuropsychiatric disorders like attention deficit hyperactive disorder (ADHD), obsessive-compulsive disorder (OCD), autism, or schizophrenia.^2^ In particular, impulsivity can be understood as a multifaceted phenomenon divided into different components: “impulsive action” as a failure in motor inhibition, “impulsive choice” as a tendency to accept small, immediate rewards over larger, delayed rewards, and “reflection impulsivity” as a sensory phenomenon whose evidence has not been sufficiently investigated.^3,4^ Inhibitory control deficit is prominent during adolescence,^5^ and some factors such as diet,^6^ substance abuse,^7^ or even sport^8^ have been proposed to affect its normal development. However, the effects of some specific diets, like the high-fat diet (HFD), high-sugar diet (HSD), or cafeteria diet (Caf), on the multifaceted nature of inhibitory control have not been fully studied.

HFD and HSD have been linked to impulsive behaviors in clinical studies.^9^ In adolescence, impulsivity and diet variables are closely related.^9−12^ Most studies aim to clarify the effects of obesity over different behaviors; however, few studies have analyzed the effects of dietary manipulation over inhibitory control. Adams et al.^13^ exposed rats to HFD and HSD. Increased motor impulsivity was found in subjects exposed to HFD assessed with the five choice serial reaction time task (5-CSRTT) compared with chow diet, while no effect in overall performance was found. Other studies analyzed choice impulsivity: no effect of HFD with 2:1 and 4:1 ratios of reinforcement was found;^14^ also, in HSD, no effect was reported.^15^ Nevertheless, Robertson and Rasmussen,^16^ who created obesogenic conditions, found an increased preference over larger and later (LL) rewards when subjects were exposed to a Caf diet. To the best of our knowledge, no studies have analyzed the effects of HFD/HSD or Caf consumption on impulsive decision-making. Dietary effects can also be detected without creating an obese phenotype.^17^ For example, some differences were seen in the microbiome when a HFD was maintained for 2 weeks. In addition, long-term effects in plasmatic IL-1β were detected after 5 months since the HFD was exposed.^6^ Additionally, some studies found a relationship between neuroinflammation and cognitive alterations.^17,18^

Furthermore, the activation of the immune system can also be secondary to diet.^19^ Some studies found an increase in pro-inflammatory molecules when HFD,^6^ HSD,^20^ and Caf^19^ are presented. Additionally, Zeeni et al.^21^ found that when rats were exposed to a chronic variable stress, no difference was found in the high-palatable diet groups; this diet was associated with a reduction response to chronic stress. In addition, Shin et al.^22^ found an increase in IL-1β in serum after generating an obese phenotype. However, the effects of these diets are not focused on the gut microbiome alone; they can also affect the mesolimbic and fronto-striatal pathways via activating dopamine (DA) neurons.^23^ Besides, some proteins such as brain-derived neurotrophic factor (BDNF) were reduced in male rats with four-week HFD while remaining intact in females.^24^ The existing relationship between BDNF and diet is still unknown. Long and acute exposure (2–8 months) to HFD modifies BDNF concentrations, while short exposure (20–42 days) does not seem to affect concentrations.^25^

Brainstem and striatum seem to be two biological targets for high-fat diet effects.^26^ Specifically, in the striatum, we can find dopamine (DA) receptors 1 and 2 (DRD1 and DRD2), which cohabitate with cannabinoid receptor 1 (CB1), which is closely related to food regulation and eating disorders like obesity or binge-like eating.^27−29^ Regarding DA, up-regulation of the DRD1 in the amygdala was found in rats exposed to HFD throughout adolescence;^30^ in the brainstem, an increase in relative expression of DRD1 and a reduced relative expression of DRD2 were detected. However, other authors^31^ found no differences in genes related to the DA system (*DRD1*, *DRD2*, *TH*, and *DAT*). In addition, no differences were present in *D1* and *D2* but in *COMT* in rats exposed to HFD; this difference was located in obesity-prone (OP) rats.^27^ One part of the mesolimbic circuit is the nucleus accumbens (Nacb). The Nacb shell motivates consumption of dietary fat in rats, and when D1Rs are inhibited in the lateral shell, a reduction in fat consumption can be seen.^32^ The endocannabinoid system is formed by CB1 and CB2 receptors. Rojo et al.^33^ found an increase in CB1 stimulation in the prefrontal cortex after 4–12 HDF consumption without an increase in receptor density, but no differences were present in chronic diet consumption (16–20 weeks). Regarding the Nacb, no differences were seen in *CB1* or *CB2* expression.^27^ BDNF levels are able to change even with 24 h of HFD consumption, finding differences between sexes. When exposure time rises, those differences between sexes are more pronounced.^24^ However, fat diets can also affect glutamatergic transmission in the hippocampus, specifically down-regulating NMDA receptors like *Grin2a* and *Grin2b*.^34^ There is some overlap between the biological targets of the HFD and the brain circuit related to inhibitory control. The fronto-striatal pathway, formed by several brain structures,^35^ is related to inhibitory control.^36^

Thus, the present study aims to investigate the putative relationship between HFD consumption at a young age and inhibitory control deficits in adulthood using premature responses as a measure of impulsivity, while perseverative responses were used as a measure of compulsivity, as well as determining which neurochemical changes may be related with those deficits. Different paradigms, such as the variable delay to signal (VDS), 5-CSRTT, delay discounting task (DDT), and rodent gambling task (rGT), were used to screen the three components of inhibitory control. Genetical analyses were performed using RT-qPCR in the frontal cortex, while metabolomics analyses were performed in stool samples using ^1^H NMR and gas chromatography coupled to a flame ionization detector (GC-FID) analyses, both collected at the last stage of the protocol. Our hypothesis is that rats exposed to HFD during a critical developmental period will show impaired inhibitory control-related measures compared with the chow-fed group. Furthermore, we expect differences in some genes related to neurotransmission in the frontal cortex. Also, we expect differences in the metabolomics profiles and fatty acids (FAs) of our groups.

## 2 Results and Discussion

### 2.1 Baseline Weight, Chow, and Water Consumption

**Figure 1:**
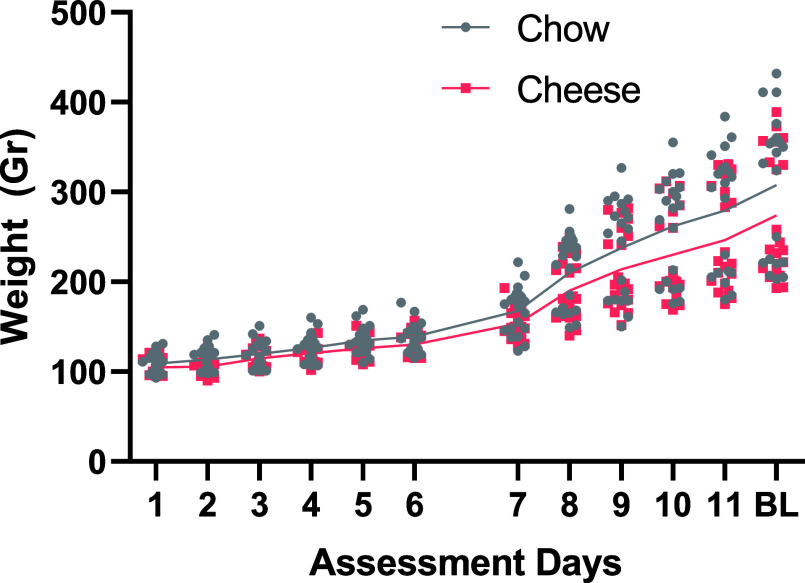
Weight evolution across all test days until baseline (BL) privation day. The period shown corresponds to the period where the rats were fed with HFD. Comparisons were performed using Bonferroni correction. Individual data is represented, and a line connects mean group values. Total *n* = 20 in each group. Numbers from 1 to 6 represent weight evolution daily, and numbers between 7 and 11 represent body weight assessed every week.

No differences were detected in body weight concerning conditions-to-be-assigned in the first day; however, the expectable sex effect (F_1,37_ = 47.299; *p* < 0.001; partial η^2^ = 0.561) and an overall day effect (F_1,148_ = 256.891; *p* < 0.001; partial η^2^ = 0.874) were present (Figure 1). No differences between groups were shown, but a predicted sex effect was shown (F_1,37_ = 54.576; *p* < 0.001; partial η^2^ = 0.596). Day*group (F_1,148_ = 4.202; *p* < 0.001; partial η^2^ = 0.102) and day*sex (F_1,148_ = 8.978; *p* < 0.001; partial η^2^ = 0.195) interactions were observed. In baseline privation day, only a sex effect existed (F_1,37_ = 304.860, *p* < 0.001, partial η^2^ = 0.892) (Figure 1).

In the baseline chow consumption test, a group (F_1,37_ = 6.823; *p* < 0.05; partial η^2^ = 0.156) and sex effect (F_1,37_ = 14.209; *p* < 0.01; partial η^2^ = 0.185) were detected. Concerning chow consumption through the assessments, a significant day (F_4,148_ = 48.402; *p* < 0.001; partial η^2^ = 0.567), sex (F_1,37_ = 78.204; *p* < 0.001; partial η^2^ = 0.679), and group effect (F_1,37_ = 256.891; *p* < 0.001; partial η^2^ = 0.874) were present. The day*group (F_1,148_ = 26.682; *p* < 0.001; partial η^2^ = 0.419) and the day*sex (F_1,148_ = 10.604; *p* < 0.001; partial η^2^ = 0.223) interactions were observed. Concerning baseline water consumption, both group (F_1,37_ = 5.293; *p* < 0.05; partial η^2^ = 0.125) and sex (F_1,37_ = 7.474; *p* < 0.01; partial η^2^ = 0.168) effects were perceived (data not shown). An overall day effect was found (F_4,148_ = 15.947; *p* < 0.001; partial η^2^ = 0.301), as well as group (F_1,37_ = 41.159; *p* < 0.001; partial η^2^ = 0.527) and sex (F_1,37_ = 20.200; *p* < 0.001; partial η^2^ = 0.353) effects regarding water consumption across all test days, but no interaction was obtained (data not shown). As expected, our results showed that both groups (cheese and chow) did not differ in terms of body weight or other physiological variables that might affect any biological or behavioral outcome. This enables us to necessarily attribute any behavioral or biological effect to reasons other than an obesogenic profile, in contrast with most of the current scientific works.

### 2.2 Motor and Cognitive Impulsivity

#### 2.2.1 Variable Delay to Signal (VDS) Training Performance and Inhibitory Control

**Figure 2:**
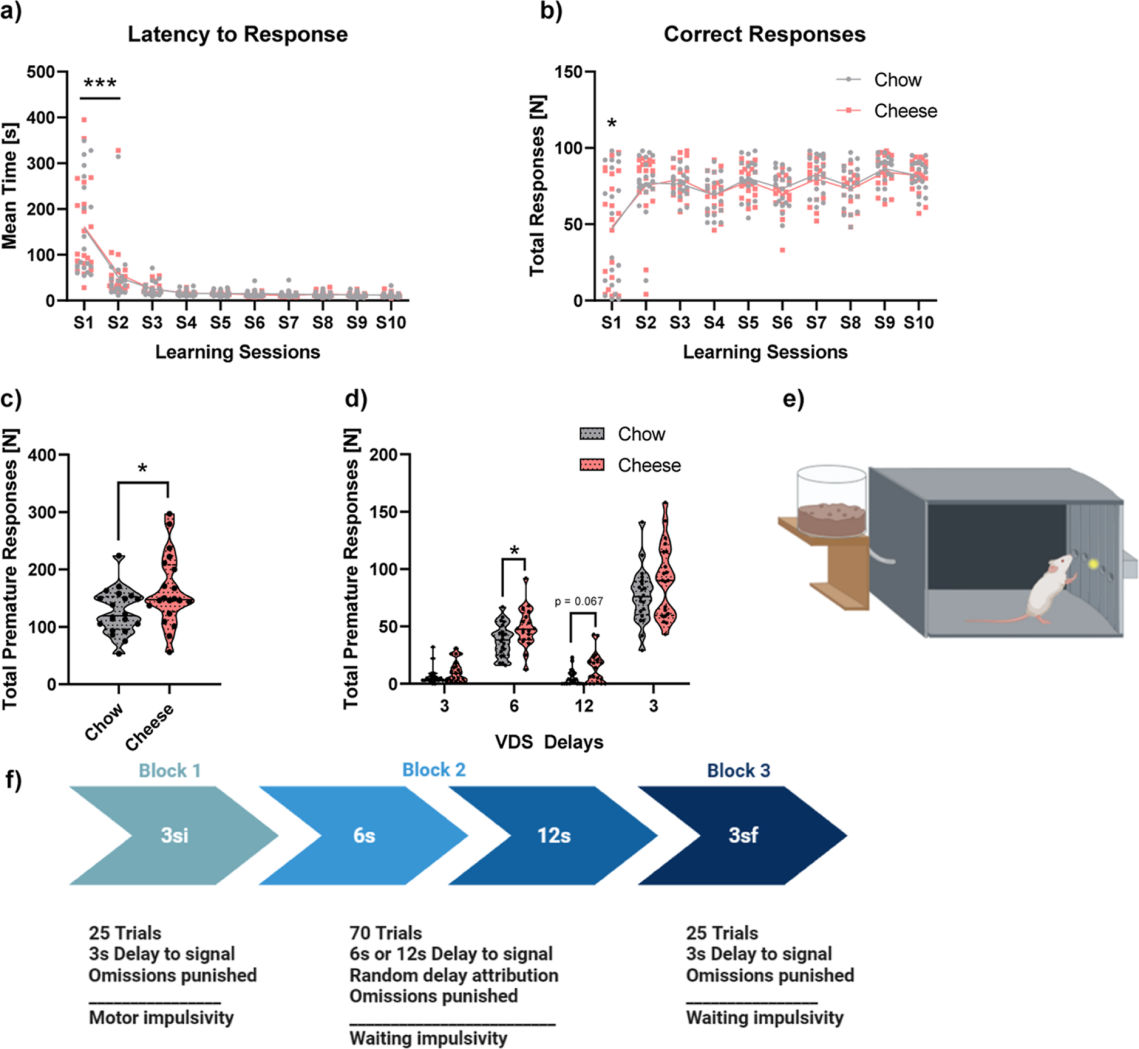
VDS training and test performance representation. In the upper part, mean latency to response across all training sessions (a) is depicted, while in (b), correct responses through learning sessions are shown. In the lower part, total premature responses (c) and total premature responses per minute in the test session are shown. In part (e), a graphical example of the operant box used is shown. Lastly, in part (f), a graphical resume of the task is shown. Total n in each group was 20. Data is represented as individual values with lines connecting means. In the lower part, violin graphs are depicted, with mean and quartiles; *p < 0.05; ***p < 0.001. Differences in graphs (a,b) correspond to session differences, not diet nor sex.

All groups showed appropriate learning, reducing total session time (F_9,333_ = 16.976; *p* < 0.001; partial η^2^ = 0.315; post hoc comparisons revealed that S1–S3 were different between them and between the other sessions; *p* < 0.05 in all comparisons) and increasing total correct responses (F_9,333_ = 5.297; *p* < 0.001; partial η^2^ = 0.125; session one was different with the other sessions with *p* < 0.001 using Bonferroni correction), reducing mean (F_9,333_ = 14.256; *p* < 0.001; partial η^2^ = 0.278; post hoc revealed that sessions 1 and 2 were different from the others with *p* < 0.001) and total (F_9,333_ = 16.976; *p* < 0.001; partial η^2^ = 0.315; post hoc comparisons revealed that sessions 1 to 3 were different from the others with *p* < 0.001) latency response, and total (F_9,333_ = 10.864; *p* < 0.001; partial η^2^ = 0.227; same post hoc results were found. Sessions 1 and 2 were different from the others with *p* < 0.001) but not mean latency to reward. No effect of sex or group was found (Figure 2a,b). Rats also tended to do fewer omissions across sessions (F_9,333_ = 13.890; *p* < 0.001; partial η^2^ = 0.273; sessions 1 and 2 had more omissions compared to the others with *p* < 0.001). A session*sex interaction was found (F_9,333_ = 2.387; *p* < 0.05; partial η^2^ = 0.061) in total session time; however, no effect of sex was evidenced.

No effect was found in premature responses: although there was an overall session effect (F_9,333_ = 8.579; *p* < 0.001; partial η^2^ = 0.188), no group or sex effects were detected. Moreover, in prematurity response rate, an overall session effect was observed (F_9,333_ = 5.651; *p* < 0.001; partial η^2^ = 0.132); no group or sex effects were found. Rats did more premature responses throughout all of their training sessions. Similarly, no effect existed in perseverative responses: although a session effect was found (F_9,333_ = 4.223; *p* < 0.001; partial η^2^ = 0.102), no group nor sex effects were detected. Rats made more perseverant responses in the first three training sessions.

#### 2.2.2 VDS Test Performance and Inhibitory Control

No effect was found in correct responses, omissions, total response latency, mean response latency, total latency to reward, or mean latency to reward. On the contrary, a strong effect of group was found in total premature responses (F_1,35_ = 4.872; *p* < 0.05; partial η^2^ = 0.122) (Figure 2c,d). When analyzing the different delays, an effect of group was detected in the 6 s delay (F_1,35_ = 4.198; *p* < 0.05; partial η^2^ = 0.107), as well as a trend close to significance in the 12 s delay (F_1,35_ = 3.885; *p* = 0.057; partial η^2^ = 0.1), but no effect was noted in the others. No sex effect was perceived in the total premature responses in any of the VDS blocks. Rats with HFD consumption tended to do more premature responses regarding total premature responses and premature responses in 6 and 12 s delay.

#### 2.2.3 Five Choice Serial Reaction Time Task (5-CSRTT) Training Performance and Inhibitory Control

**Figure 3:**
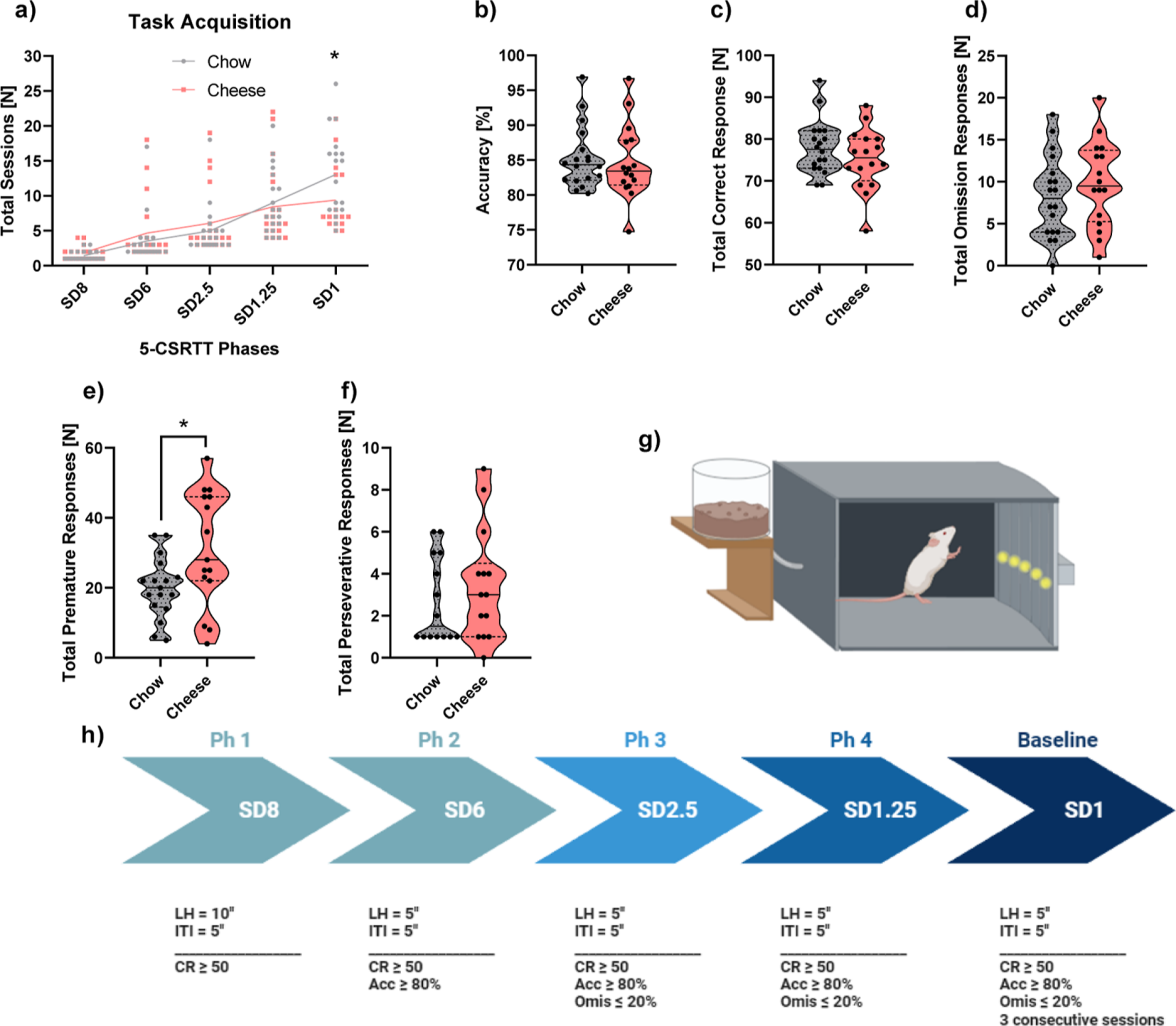
Graphical image of the 5-CSRTT learning and baseline inhibitory control measures. In part (a), total sessions to achieve the baseline condition (SD1) are shown; a line connects the mean in every session. In parts (b–d), task learning is shown as accuracy, correct responses, and total omission responses, respectively. In part (e), total premature responses are depicted, and in part (f), total perseverative responses are shown; these graphs are violin plots where the mean and quartiles are represented. In part (g), a graphical example of the operant box used is shown. Lastly, in part (h), a graphical resume of the task is shown. Individual data is represented as well as the mean ± SEM *p < 0.05. Sample sizes were as follows: *n* = 16 for cheese and *n* = 18 for chow. * refers to group differences (chow vs cheese).

All rats learned the task appropriately. No differences were found according to the sessions required to reach each criterion. However, a significant difference was observed in the total sessions required to achieve SD1 criteria (F_1,29_ = 4.989, *p* < 0.05, partial η^2^ = 0.147). The control group needed more sessions to achieve the criteria (Figure 3a). The analysis of covariance (ANCOVA) revealed no differences in any learning variables in the three consecutive sessions required to achieve SD1 criteria (Figure 3b–d). However, the ANCOVA revealed that the cheese group did more premature responses than control (F_1,31_ = 4.637; *p* < 0.05; partial η^2^ = 0.128). No effect was found for perseverative responses (Figure 3e,f).

#### 2.2.4 Delay Discounting Task

The RM-ANCOVA revealed a large delay effect across all delays (F_4,128_ = 27.691; *p* < 0.001; partial η^2^ = 0.461). No effect of group was found, nor sex effect, in any comparison. Rats showed more mean choices for the LL during the DDT (Supporting Information 1, Figure S1). All the animals chose the LL when the delay is 0 or close (5 s), but when this increases, they tend to choose the SS.

Surprisingly, we found that HFD exposure in the adolescence period seemed to affect differently depending on the impulsive subphenotype analyzed. These results are in accordance with previous studies like in Adams et al.^13^ where the diets (HFD and HSD) were administered during adulthood, while ours was administered during adolescence. Their results show that HFD leads to more premature responses compared to the other groups. Literature also supports the idea that high motor impulsivity is related to higher high-fat binge-like eating consumption,^37^ thus pointing to the possibility of the existence of a vulnerability in motor impulsivity after HFD consumption in adolescence. In addition, our findings are in accordance with Cussotto et al.,^19^ where the authors propose a relationship between diet-related factors and a myriad of maladaptive behaviors.

In regard to choice impulsivity, assessed with the delay responses in the DDT, no effect was found. Our results are in accordance with others like in Garman et al.,^14^ where a HFD was provided ad libitum for 14 days and no differences in impulsive choice were found. In addition, Narayanaswami et al.^38^ studied the breakpoint of rats exposed to a HFD for 8 weeks; they divided the sample into obesity-prone (OP) and obesity-resistant (OR) according to body weight. No difference was found between the OP and the OR groups. This long-term vulnerability seems to not affect all the impulsivity subtypes^3^ because effects were found in motor but not in choice impulsivity. Aside from the heterogeneous nature of the phenomenon, this difference might be explained by methodological variability across studies: in the study previously reported,^38^ they used data from the quartile 1 and quartile 4 (rats that gained the most weight vs rats that did not gain enough weight), while other studies used different approaches. This division has been used as a suitable method for studying diet-induced obesity (DIO),^39,40^ but, arguably, when the objective is not to directly study an obese phenotype, subtle differences in inhibitory control deficit might be difficult to identify or even fail to appear. Another explanation could be the differences regarding diet type (HFD, HSD, and Caf^16,41^) and the time that it is available.^42−44^ Steele et al.^44^ found that rats preferred the SS more than the LL in the DDT when they performed the task when off diet. However, their choice changed, and they preferred the LL more than the SS when on HFD.

Regarding compulsivity, our results show no deleterious effect of HFD. No differences were observed in perseverative responses on VDS or 5-CSRTT. These results are in accordance with previous observations;^13,38^ however, some authors have reported differences in the marble burying test^42,45^ developing DIO. These results were also observed in monkeys, where they showed more perseverative responses in a reversal learning task when HFD was consumed ad libitum.^46^ More research is needed to fully understand the relationship between compulsivity and diet. Kakoschke, Aarts, and Verdejo-Garcia^47^ exposed that this relationship could be explained by contingency-related cognitive flexibility, task/attentional set-shifting, attentional bias/disengagement, and the results of habit learning.

### 2.3 Rodent Gambling Task

**Figure 4:**
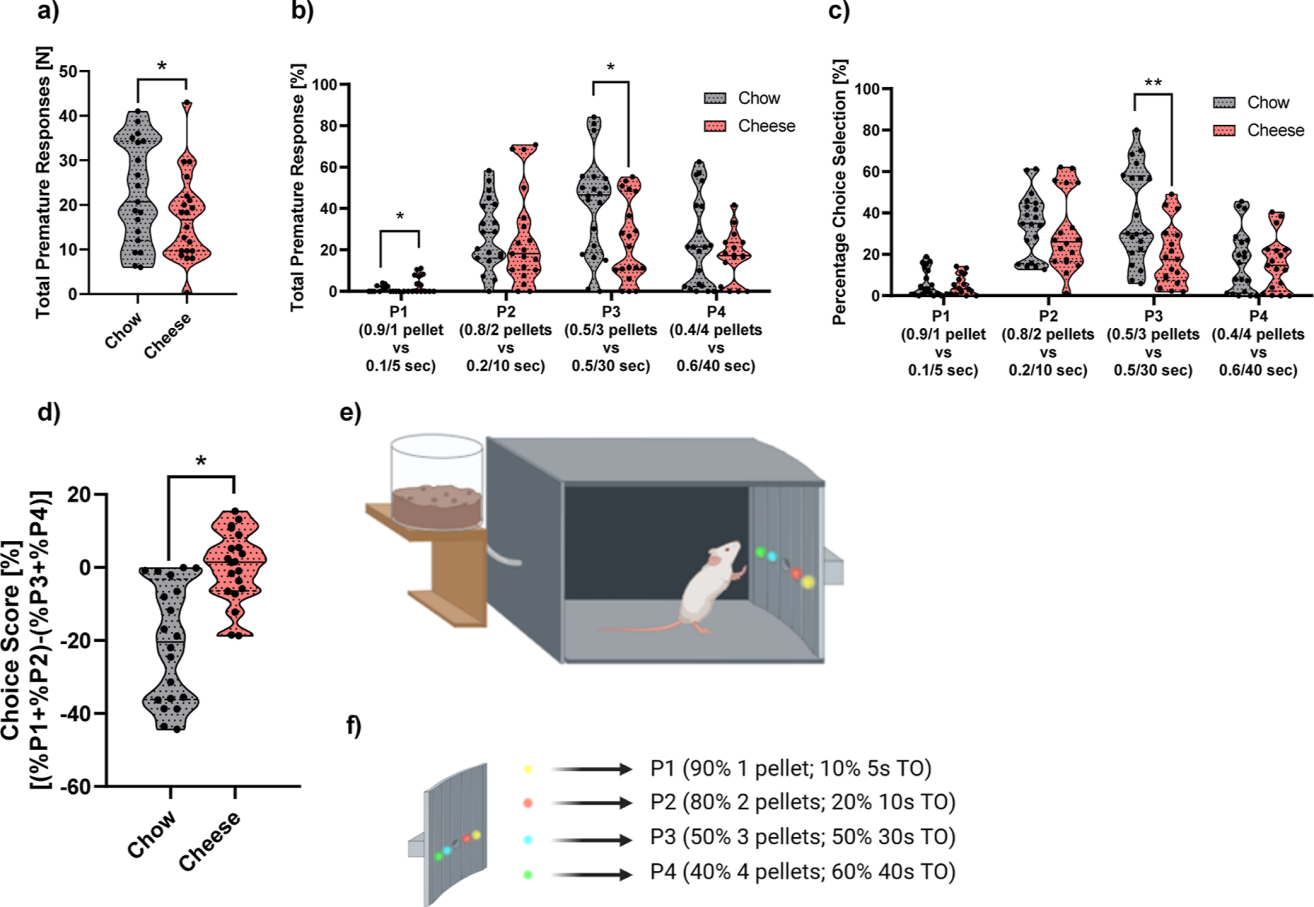
Graphical representation of rGT’s variables. In the upper part, total premature response (a) and total percentage premature response (b) are depicted. In addition, percentage choice selection (c) and percentage choice score (d) are shown. Sample size is as follows: in graph (a), 19 for chow and 20 for cheese; in part (b), P1 (*n* chow = 14; *n* cheese = 15), P2 (*n* chow = 19; *n* cheese = 19), P3 (*n* chow = 20; *n* cheese = 19), and P4 (*n* chow = 20; *n* cheese = 15). In graph (c), P1 (*n* chow = 20; *n* cheese = 16), P2 (*n* chow = 18; *n* cheese = 18), P3 (*n* chow = 20; *n* cheese = 17), and P4 (*n* chow = 19; *n* cheese = 17); in part (d), it is 20 in each group. Individual plots were shown when possible; the mean ± SEM is depicted in every image. *p < 0.05, **p < 0.01.

All rats learned the task appropriately. A significant effect of group was found in percent premature responses (F_1,35_ = 8.381; *p* < 0.01; partial η^2^ = 0.235); sex also reached significant levels (F_1,35_ = 7.462; *p* < 0.01; partial η^2^ = 0.176), but its interaction was not significant. The cheese group did fewer premature responses than chow, and males did higher responses compared with females (Figure 4a).

The premature responses were also analyzed for every possible response. We found a trend to significance in P1 (F_1,26_ = 3.597; *p* = 0.069; partial η^2^ = 0.122) without any sex effect and a group significance in P3 (F_1,35_ = 4.709; *p* < 0.05; partial η^2^ = 0.119), being the cheese the less impulsive, with a trend to significance in the sex (F_1,35_ = 3.879; *p* = 0.057; partial η^2^ = 0.100; Figure 4b). The cheese group showed less percent premature responses (*M* = 25.272; SD = 19.233) than the chow group (*M* = 40.546; SD = 23.058), and females (*M* = 25.978; SD = 18.569) were higher than males (*M* = 39.839; SD = 23.722). Same results in P3 were found in percent choice selection for the group (F_1,34_ = 7.357; *p* < 0.01; partial η^2^ = 0.178; Figure 4c). Sex concentrations did not reach significant levels. No effect was detected for perseverative responses in total or in percent.

In addition, a significant difference was evidenced in percent reinforced trials (F_1,30_ = 6.365; *p* < 0.05; partial η^2^ = 0.175), where cheese got more reinforced trials than control (cheese; *M* = 64.214; SD = 7.113; chow; *M* = 58.279; SD = 3.232). A trend for the group was observed in total latency (F_1,32_ = 4.018; *p* = 0.054; partial η^2^ = 0.112), with cheese being the group with higher latencies (cheese; *M* = 59412.627; SD = 27095.943; chow; *M* = 44389.019; SD = 12350.852). No difference was detected for punished trials. To end up with, we detected a strong effect of group on the percent choice index (F_1,37_ = 5.523; *p* < 0.05; partial η^2^ = 0.130). The cheese group had a higher index than chow (Figure 4d).

Intriguing long-term effects of HFD consumption were seen in decision-making assessed with the rGT in the present study, where HFD rats seemed to be more conservative in their responses. Apparently, they tend to cope with less risk by making more premature responses in the most rewarded and less in the 50%-reward and 50%-punish options. This result is in discrepancy with the reported motor impulsivity tasks such as the 5-CSRT task and VDS. Both tasks assess impulsivity/compulsivity with a light-dependent response. The subject must respond to the light making a nosepoke where it has been shown for a specific period of time to receive a reward.^48^ However, even though rGT is also a light-dependent task, it assesses impulsive decision-making, not motor impulsivity. Some authors have considered the rGT as a waiting impulsivity task;^49,50^ however, the task’s objectives can lead to a different consideration of its nature. In the 5-CSRTT, the animal only has to respond to a light in order to get a reward, while in the rGT, the animal must follow a specific decision making process in order to choose the option that fits best (see Supporting Information 1, Behavioral Analysis). These differences might root in the rGT^5,51^-CSRT task^48^ distinct methodologies. Moreover, we also found a difference in the percentage of choice-selection on P3 and in the choice-selection index, showing that HFD was a more conservative choice than control. Regarding clinical models, Navas et al.^52^ found that obese individuals made riskier choices than controls, showing a possible link between risky decision-making and obesity. These results may seem contradictory; however, the influence of diets on decision-making may be explained through two possible options. First, a reinforcement devaluation process could be present in our measure. Cycled-Caf diets showed reduced liking behavior to a sucrose solution at 2% (our test diet is 3% sucrose).^53^ Second, the foraging strategies changed with the caloric value of the different diets. The influence of palatability on motivation to respond for noncaloric food and caloric food is different if the rats are food-deprived or not.^54^

### 2.4 RT-qPCR Gene Expression

**Figure 5:**
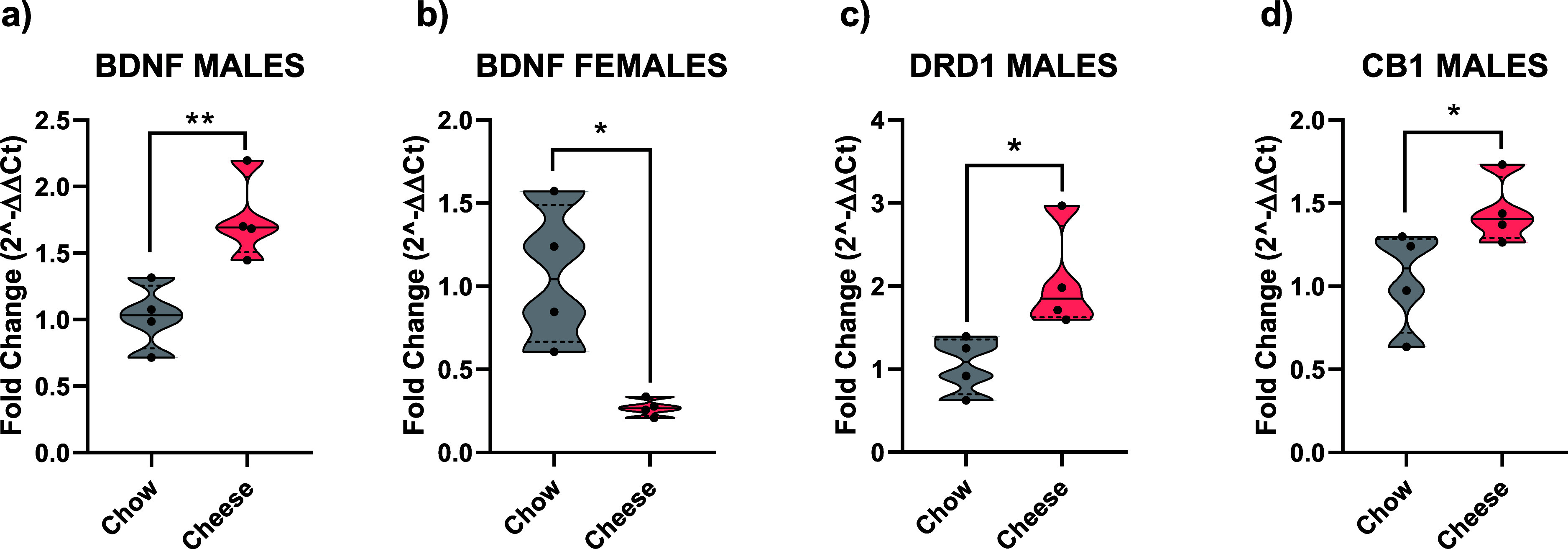
Visual representation of the genes that achieved significant differences in the *t*-students. *p < 0.05; **p < 0.01. Total *n* = 4 in each group.

Independent *t* tests were performed according to sex to be able to use the chow groups (chow-male and chow-female) as a control. In the case of male rats, differences were found in *BDNF* fold change (*p* < 0.01; *d* = −2.593), in *CB1* (*p* < 0.05; *d* = −1.613) and in *DRD1* (*p* < 0.05; *d* = −2.018) (Figure 5a–d). No differences were detected in *DRD2*, *GAD1*, *TNF-*α, and *TYRO* fold change in PFC. Regarding female rats, differences were obtained in *BDNF* fold change (*p* < 0.05; *d* = 2.620), but no differences were noticed in *CB1*, *DRD1*, *DRD2*, *GAD1*, *TNF-*α, or *TYRO* (Supporting Information 1, Figure S2).

Our brain gene expression results showed long-term alteration in fold change in some genes related to two main neurotransmitter systems related to food regulation. Long-term effects were detected in *DRD1* expression only in males, while no differences were seen in females. This result is in accordance with previous results in other brain areas like the brainstem^26^ and amygdala,^30^ two components of the mesolimbic pathway.^55^ The endocannabinoid system was also affected. Satta et al.^56^ found reduced levels of anandamide in the frontal cortex and amygdala but higher levels in Nacb. On the other side, 2-arachidonoyl glycerol was increased in HCC. This system has been proposed to be closely related to food rewards and associated with the DA system.^57^ Moreover, differences in endocannabinoid tone are found in other mesolimbic pathways such as the amygdala, caudate-putamen, and hippocampus.^56,57^

Some studies have found an effect of HFD on HCC morphology and function.^34,58,59^ To the best of our knowledge, no studies have reported BDNF dimorphism coexisting with inhibitory control deficits. While BDNF expression increased in males, it decreased in females. It seems that females are more vulnerable than males in terms of their long-term vulnerability after HFD consumption in adolescence. Some studies have shown that HFD and Caf are associated with reduced levels of BDNF in the HCC.^57^

### 2.5 CORT, Leptin, and TNF-α Serum Level Analysis

**Figure 6:**
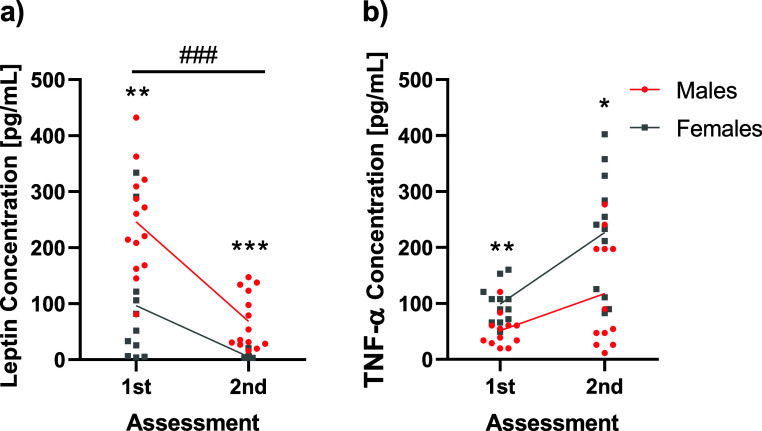
Graphical representation of leptin (a) and TNF-α (b) in serum between the two assessments. Individual values are shown in every assessment and in every condition. Sample size is as follows: *n* = 14 for males and *n* = 13 for females in graph (a) and *n* = 12 for graph (b). *p < 0.05, **p < 0.01, ***p < 0.001, ###*p* < 0.001. * represents group differences, while # represents assessment differences.

Regarding leptin, the RM-ANCOVA revealed differences between time measures (F_1,22_ = 19.030; *p* < 0.001; partial η^2^ = 0.464). The covariable also reached significance levels (F_1,22_ = 17.095, *p* < 0.001: partial η^2^ = 0.437); when sex was included as an intrasubject factor, differences were also detected (F_1,21_ = 13.734; *p* < 0.001; partial η^2^ = 0.395). Males and females were different in leptin concentration in the first assessment (F_1,24_ = 11.587; *p* < 0.01; partial η^2^ = 0.345) and in the second assessment (F_1,24_ = 18.975; *p* < 0.001; partial η^2^ = 0.463) (Figure 6a).

TNF-α levels were equal between the assessments and groups. In this RM-ANCOVA, the covariance reached significance levels (F_1,22_ = 13.309; *p* < 0.001; partial η^2^ = 0.377). When sex is included as an intrasubject factor, it also reached significant differences in the first assessment (F_1,24_ = 11.303; *p* < 0.01; partial η^2^ = 0.350) and in the second assessment (F_1,24_ = 4.625; *p* < 0.05; partial η^2^ = 0.180) (Figure 6b).

Even though we did not find any effect of HFD on TNF-α levels, the other results seem to strengthen our findings of altered phenotype without differences in body weight, thus pointing to a vulnerability mechanism which is not related to body composition or obesogenic conditions.^60,61^ The potential explanation of behavioral abnormalities being related to neuroinflammation cannot be confirmed either since no differences were observed in TNF-α in serum or in RTqPCR for TNF-α, thus demanding further investigation.

### 2.6 NMR and GC-FID Metabolic Profiles

On the one hand, the NMR analyses showed several essential and nonessential amino acids (valine, leucine, isoleucine, alanine, threonine, glutamate, aspartate, and glycine). Furthermore, organic acids (succinate, gumarate, formate, choline, betaine, glucose, bile acids, and short-chain fatty acids [SCFAs; acetate, propionate, and butyrate]) that serve as an indicator of microbial and metabolomic processes were detected. All NMR-detected metabolites are explained in the Supporting Information 2 (Table 1).

**Table 1:** Fold Changes and *p*-Values for Differential NMR Peaks between Chow and Cheese Groups in Male and Female Feces

	fold change (chow vs cheese)	log_2_(FC)	*p*-value
Male			
2.66 (aspartic acid)	1.20	0.26	0.037
2.81 (aspartic acid)	1.19	0.25	0.023
Female			
0.88 (fatty acids except *n* – 3)	1.21	0.27	0.020
4.59 (glucose)	0.85	–0.24	0.007
3.39 (glucose)	0.82	–0.28	0.021
3.46 (glucose)	0.86	–0.21	0.021
5.20 (glucose)	0.75	–0.41	0.024
2.88 (hydrocinnamic acid)	0.89	–0.16	0.034
3.63 (glycerol)	1.15	0.20	0.041

**Figure 7:**
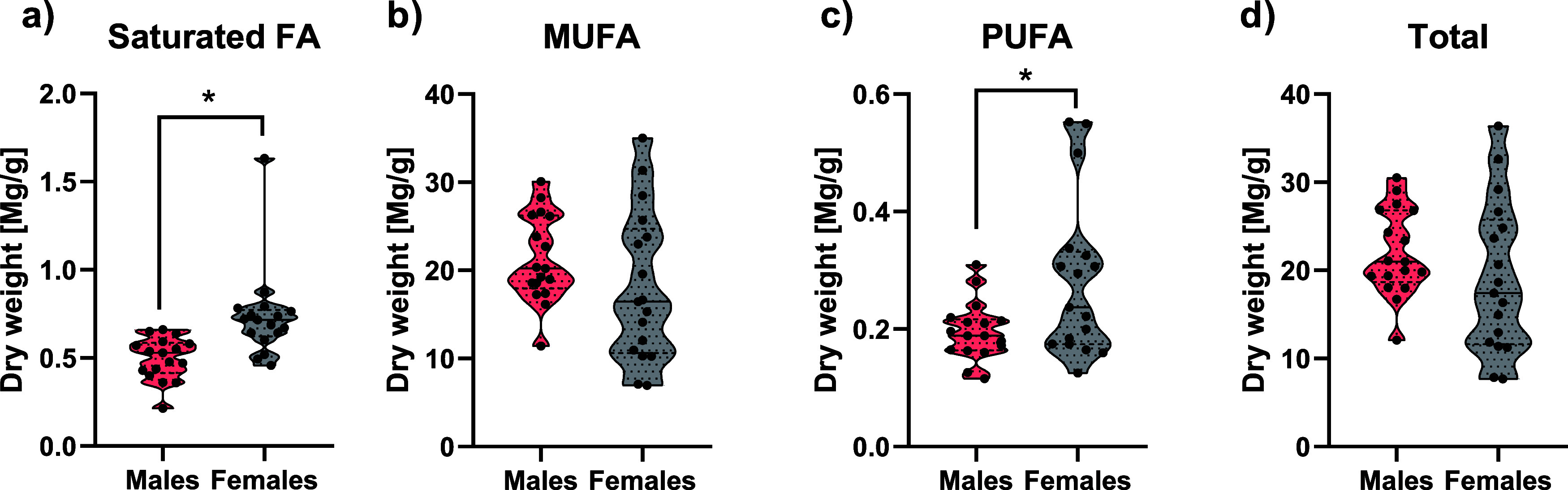
Boxplots of FA concentrations, distributed in saturated, monounsaturated, and polyunsaturated FAs and total FAs and detected by GC-FID in male and female feces. Differential concentrations determined by an unpaired *t*-test (*p* < 0.05) between male and female feces were marked with *. Total *n* in each group is 17.

**Figure 8:**
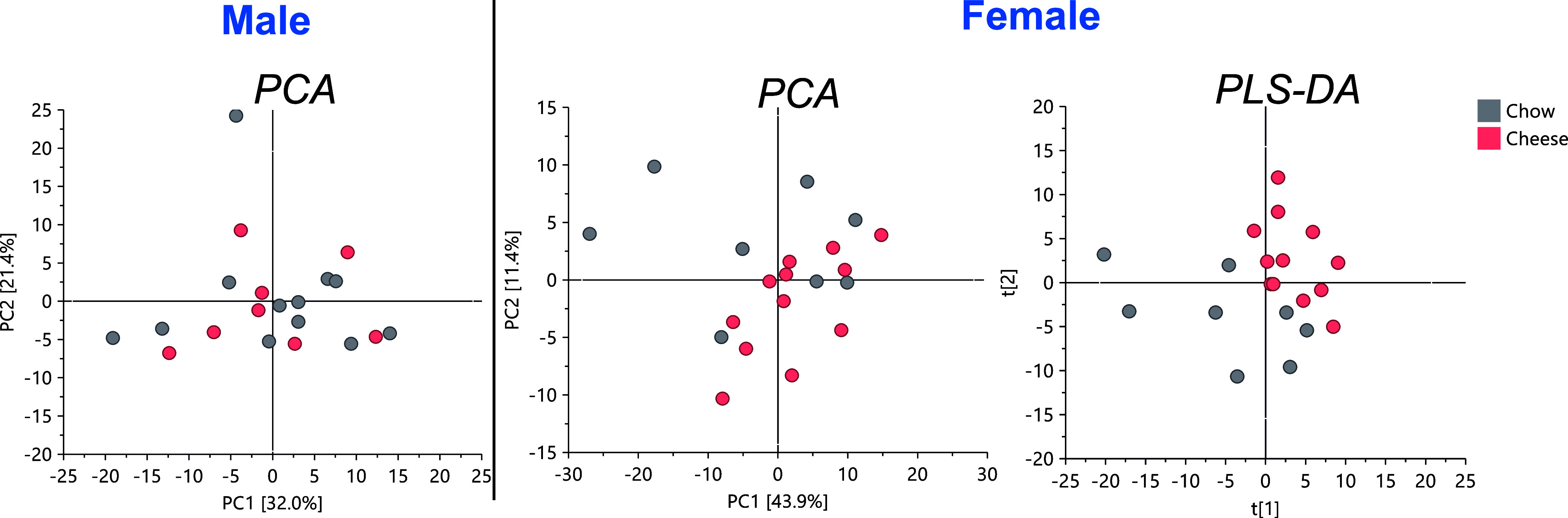
PC1/PC2 PCA models applied to ^1^H NMR data of fecal extracts from male and female rats. A valid PLS-DA model showing discrimination between chow and cheese groups was only found in female fecal extracts (R^2^X = 0.61, R^2^Y = 0.994, *Q*^2^ = 0.514, CV-ANOVA = 0.22). Total n in the male PCA is as follows: chow (*n* = 12) and cheese (*n* = 8); female PCA n is as follows: chow (*n* = 8) and cheese (*n* = 12); female PLS-DA *n* is chow (*n* = 8) and cheese (*n* = 11).

On the other hand, the GC-FID analyses revealed 13 fatty acids (FAs), of which 7 were saturated (palmitic, capric, and lauric acid the major ones), 3 were monounsaturated (eicosenoic acid the most frequent, followed by oleic and vaccenic acids), and 3 were polyunsaturated fatty-acid (PUFA) chains from linoleic acid. Specifically, saturated FAs and PUFAs were found in a higher ratio in female stool samples (Figure 7a–d; Supporting Information 2 (Table 2)). Furthermore, other acids (*cis*-9,12-hexadecatrienoic and *cis*-6,9,12-hexadecatrienoic) were present in female stool samples, while they remained undetected in male stool samples (Figure 8).

**Table 2:** Fold Changes and *p*-Values for Differential NMR Peaks between Chow and Cheese Groups in Male and Female Feces for FAs

	fold change (chow vs cheese)	log_2_(FC)	*p*-value
Male			
18:1*n*9 (oleic acid)	1.3	0.38	0.045
Female			
12:00 (lauric acid)	2.0	0.99	0.032

**Figure 9:**
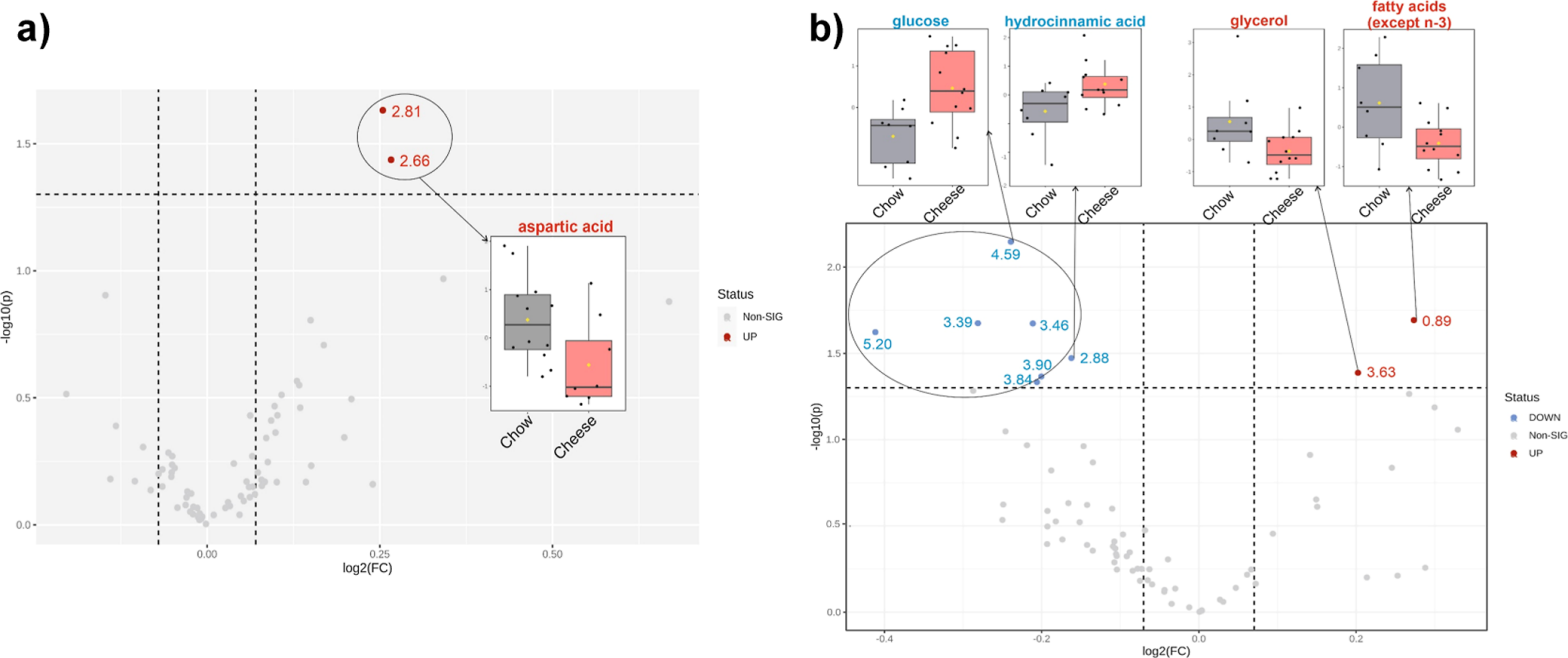
Volcano plots for (A) male and (B) female feces showing the differential metabolites between chow and cheese groups, displaying in ordinate the level of significant difference (−log10(*p*-value)) and in the abscissa the expression fold change (log_2_FC). All statistically valid FC of metabolites were considered according to Wilcoxon rank-sum tests (*p* < 0.05). The male volcano plot has *n* = 12 for chow and *n* = 8 for cheese, while the female volcano plot has *n* = 8 for chow and *n* = 12 for cheese.

NMR peaks were deeply analyzed with univariate analysis, showing that there were some metabolites with significant fold changes between the chow and cheese groups for male and female groups. Those results are shown in Table 1. Furthermore, volcano diagram analyses showed an increase of aspartic acid and both FAs (assigned to the CH_3_ terminal of all FA chains except those from omega-3 FA) in male stool samples, while glycerol was in female feces for the chow group. In addition, glucose and hydrocinnamic acid were decreased in the chow group in female stool samples (Figure 9b).

**Figure 10:**
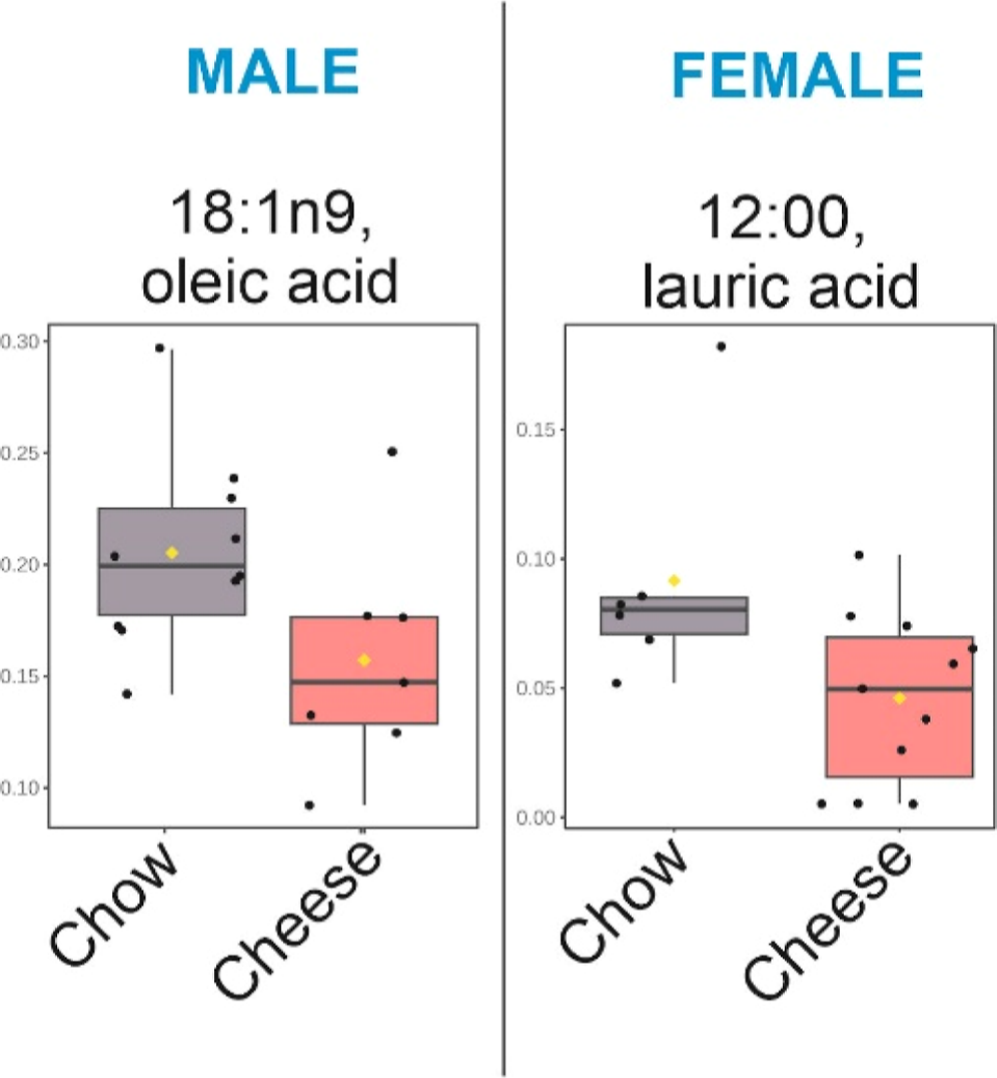
Boxplots for differential FA concentrations between chow and cheese groups in male and female feces by GC-FID. Oleic acid n is as follows: chow (*n* = 10) and cheese (*n* = 7); lauric acid *n* is chow (*n* = 6) and cheese (*n* = 11).

The NMR spectra showed that, in male stool samples, oleic acid was increased in the chow group despite FA being not a discriminating biomarker (Figure 10 and Table 2). This result may be due to the reduced fold change obtained, which might be difficult to detect in NMR spectra where all FA chains overlap. In female samples, a 2-fold increase in lauric acid (a saturated FA) was present in the chow group. So, it is possible to conclude that this saturated FA was responsible for the discriminatory difference in the integral of the CH_3_ terminal peak from all FAs except *n-3* FA (that include saturated FAs) previously found by NMR between cheese and chow groups.

Our results are in accordance with other findings in previous research. Specifically, Abreu et al.^62^ found that compulsive-like rats, selected using the schedule-induced polydipsia,^63^ had higher fatty acid levels (but not saturated) than the control group. In addition, glucose and glycerol levels were reduced in the compulsive-like group. Merchán et al.^64^ found a similar pattern: lower levels of glucose in the serum of the high compulsive-like group. Glucose influences decisions by indicating the body’s energy status rather than merely acting as a source for replenishing cognitive processing efforts.^65^ Wang and Huangfu^66^ tested this idea by giving some participants glucose at different dosages and asking them to complete an intertemporal delay discounting task. Their results revealed a negative correlation between the rate of delay and blood glucose levels. This effect was only present in the glucose-feed group, suggesting that the behavioral effects were related to hunger reduction. Our results may go in accordance with their idea: a predictive mechanism of the glucose-insulin system for managing both metabolic and behavioral aspects of acquiring and distributing resources. Furthermore, Hui et al.^67^ showed that women with gestational diabetes (with the glucose-insulin system unbalanced) have problems with adaptations to dietary management in a limited time period. Little information can be found about the relationship between the FA and decision-making. In a randomized control trial, Antypa et al.^68^ detected that after supplementation with omega-3, the participants made fewer risk-aversive decisions than the placebo group. Summarizing this information, glucose may affect decision-making by modifying the strategies required to manage metabolic and behavioral outcomes. However, the relationship between FAs and inhibitory control deficits is still unknown. As Agostini et al.^69^ claimed in their review, the association between ADHD symptoms and n-3 FA (omega 3) represents a consistent finding among observational studies, but less evidence that links the other type of FA with ADHD symptoms can be found. Pase et al.^70^ showed that, after a HFD exposure in an early development period, a hyperactive behavior could be seen after maturation. Our results complement those by Pase et al.:^70^ we found that, after an adolescent HFD consumption, higher levels of FA were present in the exposed group, which revealed higher ADHD-like behavior (increased motor impulsivity and risky decision-making). However, most of the research found that feeding supplementation with omega 3 reduces ADHD-like behaviors in rodents and in humans.^69^ Our results may also be related to this pattern; no effect of HFD was found on omega-3 FA, but the rest were affected (by sex or diet).

## 3 Conclusions

In summary, we found that HFD consumption during a critical developmental stage is related to long-term deficits in inhibitory control, specifically in impulsive-like behaviors. Moreover, this long-term vulnerability seems to differentially impact the different subcomponents of the inhibitory control. In addition, HFD consumption affects PFC, thus dramatically interfering with the mesolimbic pathway function. Furthermore, HFD exposure modifies the gut metabolic profile, affecting FA, glucose, and other compounds related to different neurobehavioral outcomes. To the best of our knowledge, this is the first study that has found long-term effects in impulsive behavior when HFD consumption takes place in adolescence. More research is needed to disentangle the specific mechanisms underlying these intriguing effects. To conclude, we have shown that exposure to a HFD in a critical developmental period (adolescence) can create a long-term vulnerability in adulthood in all of the domains analyzed. Moreover, it is worth noting the divergent effect that the HFD has on impulsivity measures, seeming to impact some specific domains while leaving unchanged others. The relationship between HFD consumption and decision-making demands further research.

## 4 Materials and Methods

### 4.1 Subjects

40 Wistar rats (20 male and 20 female; ENVIGO, Barcelona, Spain) were used in the present study. They arrived at the lab on postnatal day 21 (PND21) and were housed in groups of four rats per cage (57 × 35 × 20 cm) at 22 ± 1 °C and under a 12:12 h inverted light–dark cycle with lights off at 09:00. Environmental enrichment (PVC and wooden blocks) was added to their home cages. Food and water were provided ad libitum. At arrival, 11 days of habituation to the environment took place, after which handling was performed daily along with body weight gain and food and water intake assessment (described in baseline consumption assessment). After this, body weight control was performed once per week. Assignment to each of the experimental groups (chow or cheese) were done randomly. Once the diet manipulation was over (PND77), rats were fed ad libitum until PND96. Food deprivation started with the objective of achieving 85% of their weight at PND96 until sacrifice PND298.

All the procedures were conducted in agreement with Spanish Royal Decree 55/2013 on the protection of experimental animals and European Directive (2010/63/EU) and were approved by the University of Almera Research Committee. All the researchers show commitment to the three Rs principle.

### 4.2 Experimental Design

**Figure 11:**
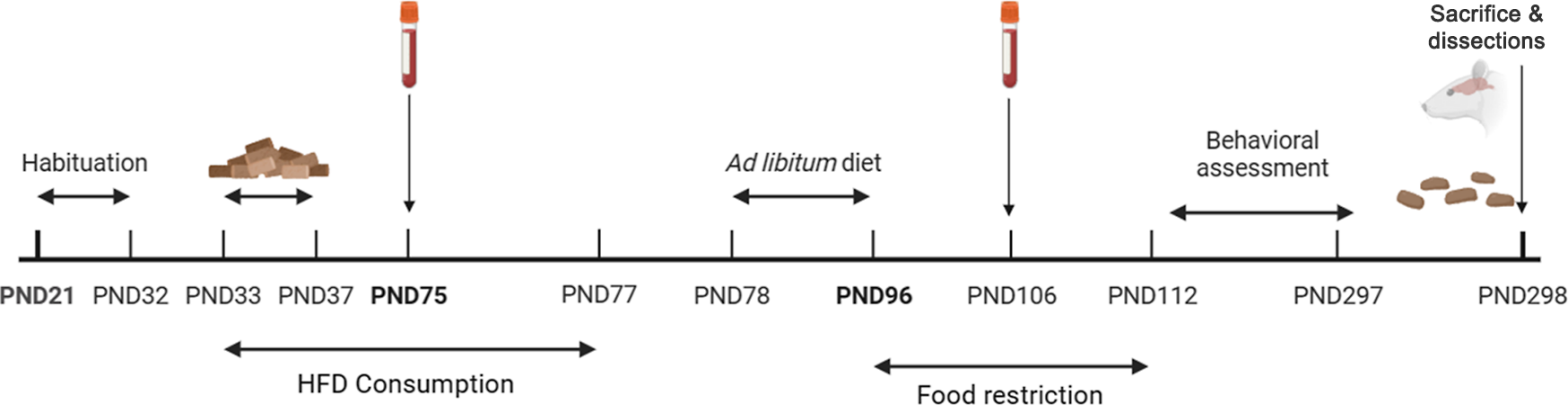
Experimental and behavioral procedures illustrated in a timetable. After arriving at postnatal day 21 (PND21) and a period of habituation to the laboratory (PND21–32), a basal chow test consumption between PND33 and PND37 was conducted, and between PND33–77, the high-fat diet (HFD) was given to the rats. Within the HFD time, a blood collection test was done at PND75. After the HFD was complete, an ad libitum diet was given from PND78 until PND96. From PND96 until PND112, the diet was restricted until rats achieved 85% of their weight. Another blood collection was done at PND106. The behavioral assessment followed, starting at PND112 until PND297. At the end, rats were sacrificed at PND298, where brains and stool samples were collected.

Subjects were assigned to one of two experimental conditions: chow (*n* = 20) or cheese (*n* = 20) [male-cheese, *n* = 12; male-chow, *n* = 8; female-cheese, *n* = 8; female-chow, *n* = 12]. After 11 days of habituation to the laboratory (PND21–PND32), a test of basal consumption (PND33) was performed, after which exposure to HFD began (PND33–PND77). On the initial days of dietary manipulation, a test of HFD-related consumption was performed (PND34–37). Once in adulthood, dietary manipulation ended, and after 2 weeks of stabilization and a chow-based diet, food restriction was gradually performed until animals reached 85% of their previous body weight. From PND96, a normocaloric diet was used for maintaining the subjects at 85%. Behavioral assessment started at PND112 and finished at PND297 (Figure 11). Brain dissection and stool collection were performed at PND298.

### 4.3 High-Fat Diet (HFD) Protocol

In accordance with previous literature,^71^ a highly caloric, high-fat diet was provided based on commercial cheesecake (Postres Reina, Caravaca de la Cruz, Spain), with the following energetic and macronutrient specifications (per 100 g): 800 kJ/191 kcal as an energetic value (10%); 9.9 g of fats (14%), of which 6.1 saturated (31%); 21.4 g of carbohydrates (8%), of which 18.5 g (21%) of sugar; 4.1 g of proteins (8%); and 0.2 g of salt (2%). Daily quantities were chosen based on the recommendations by Leigh et al.,^72^ with a total of 1 kJ per g for each rat, and adjusted based on body weight evolution. Laboratory diets remained ad libitum for both groups during dietary manipulation.

### 4.4 Behavioral Assessment

Task organization was set in order to not interfere with each other. To prevent this from happening, behavioral assessment started with a light cue task (variable delay to signal; VDS) followed by a lever cue task (delay discounting task; DDT); both tasks are different in response (light response in VDS and lever-press response in DDT). After these tasks, the 5 choice serial reaction time task (5-CSRT), a light response task, was conducted; afterward, the rodents gambling task (rGT) was conducted. A minimum of 1 week washout was left between tasks. All the behavioral assessment was performed during the dark/active phase.

#### 4.4.1 Apparatus

The behavioral tests were performed in one set of six and another of eight operant-conditioning chambers (MED Associates) measuring 32 cm long × 25 cm wide × 34 cm high, with stainless-steel grille floors. Neither the modules/wall panels used for VDS, DDT, and 5-CSRT (set 1, six chambers) were changed between tasks. A 5-CSRT task panel wall, consisting of five contiguous square holes (2.5 cm), a height of 2 cm above the grilled floor, and 2.2 cm deep, was used for VDS and 5-CSRT; a detailed description can be found in Moreno et al.^63,73^ For DDT, two retractable levers were available in the opposite wall of the operant chambers; details are available in Cardona et al.^74^ In order to minimize any possible interference between tasks, the order was as follows: VDS (nosepoke)—DDT (lever press)—5-CSRTT (nosepoke)—rGT (nosepoke). Each task commenced 1 week after the previous one. The scheduling and recording of experimental events were done by a Med PC computer and specific commercial software (Cibertec SA, Spain). Specific details of each task can be found in the Supporting Information S1.

### 4.5 Biochemical Analyses (RT-qPCR and ELISAs)

Blood samples were collected at two different moments. First, while in HFD at PND75; second, before behavioral analyses (PND106). After the completion of all tasks, all animals were deeply anesthetized with isoflurane and sacrificed with fast decapitation. Prefrontal cortex and nucleus accumbens were quickly dissected and stored separately in RNase-free tubes (1.5 mL). Also, stool samples were recollected from each rat. All samples were immediately frozen to avoid RNA degradation. All materials required were autoclaved and cleaned with RNase ZAP (Invitrogen). Samples were stored at −80 °C until use. RTqPCR was performed in the prefrontal cortex, and ELISA’s analyses were conducted with serum. The specific procedures for each biochemical assessment can be found in the Supporting Information S1.

### 4.6 ^1^H NMR Analyses

The detailed protocol that was used for the ^1^H NMR analyses can be found in Supporting Information.

### 4.7 GC-FID Analysis

The fatty acid profile and content in 10 mg of each fecal sample were determined by gas chromatography (Agilent Technologies 6890 N Series Gas Chromatograph, Santa Clara, CA, USA) after direct transesterification.^75^

The fatty acid profile and content were determined as previously described.^75,76^ Briefly, 10 mg of freeze-dried fecal sample was mixed with 1 mL of hexane and 0.125 mg of nonadecanoic acid (19:0) as an internal standard (Sigma-Aldrich, St. Louis, MO, USA). For direct transesterification, 1 mL of an acetyl chloride/methanol solution (1:20 v/v) was added. The reaction was conducted at 105 °C for 20 min. After the mixture was cooled to room temperature, 1 mL of water was added, and the mixture was agitated and centrifuged. This formed a biphasic system with the upper hexane layer containing the fatty acid methyl esters (FAMEs) derived from the fecal fatty acids. Qualitative analysis of these FAMEs was performed by a comparison of retention times of fatty acids in chromatograms with those in a commercial standard mixture from Matreya (Pleasant Gap, PA, USA). The gas chromatography coupled to a flame ionization detector (GC-FID) system was an Agilent Technologies 6890 N Series Gas Chromatograph (Santa Clara, CA, USA) with a capillary column of fused silica OmegaWax (0.25 mm × 30 m, 0.25 μm standard film, Supelco, Bellefonte, PA). Nitrogen was used as the carrier gas with a flow rate of 58.1 mL/min and a split ratio of 1:40. The injector and detector temperatures were set to 250 and 260 °C, respectively. The oven temperature was initially held at 150 °C for 3 min and then programmed to increase to 240 °C at a rate of 7.5 °C/min, where it was maintained for 12 min. Further details can be found in the protocol described in greater detail by Rodríguez-Ruiz et al.^75^

### 4.8 Statistical Analyses

Baseline weight gain was analyzed by a one-way analysis of covariance (ANCOVA), with group (chow vs cheese) as the between-group factor and sex (male vs female) as the covariable. Body weight evolution during the consumption test was analyzed by a two-way repeated measures (RM) ANCOVA, with group as the between-groups factor, day^1−5^ as the within-subjects factor, and sex as the covariable. Baseline water and chow consumption were analyzed by a one-way ANCOVA, with group as the between-groups factor and sex as the covariable. The consumption test was analyzed by a two-way RM-ANCOVA, with group as the between-groups factor, day as the within-subjects factor, and sex as the covariable. Behavioral assessment was analyzed by a two-way RM-ANCOVA (for the learning) and one-way ANCOVA (for the test) in the VDS with group and session as a between- and within-subjects factor, respectively. In the DDT and 5-CSRTT, a two-way RM-ANCOVA was conducted with group as a between factor and delays (DDT) or stimulus durations (5-CSRTT) as a within factor. In the rGT, a one-way RM-ANCOVA was conducted with group as a between factor. RT-qPCR results were analyzed with *t*-test between groups in both sexes with group as a between factor. ELISA results were analyzed via a two-way RM-ANCOVA with group as a between-subjects and time assessment as a within-subjects factor. In all ANCOVAs, sex was set as the covariable. If this covariable reached significant levels, a split analysis was conducted in order to fully understand the data dynamics. Post hoc analyses were performed, when necessary, with Bonferroni corrections. Outlier values were calculated with the GraphPad Prism tool and removed if present. Statistical significance was set at *p* < 0.05, and effect size is reported when appropriate: partial eta-squared values are reported and considered as small (0.01), medium (0.06), or large (0.14) following Cohen’s (1988) recommendations. All analyses were carried out using Statistica software (Statsoft, version 6.0) and JASP^©^ software (University of Amsterdam, version 0.14.1). Graphs were created using GraphPad Prism (San Diego, California, USA) v8.0.0, while images were designed using Biorender.

For NMR data, multivariate data analysis was performed on the obtained data set using SIMCA-P software (v. 17.0, Umetrics). Exploratory and unsupervised analysis as principal component analysis (PCA) and supervised models as partial-least-squares discriminant analysis (PLS-DA) were applied by scaling data to unit variance. Scores and loading plots were generated for both models. PLS-DA models were validated by means of their goodness-of-fit (*R*^2^) and goodness-of-prediction (*Q*^2^) cumulative values, together with the CV-ANOVA parameter validation (at the level of significance of *p* < 0.05), to test the accuracy of the model. Loadings containing important metabolites for predictive models were evaluated by generating the variable importance in projection (VIP) plot and selecting those superior to 1. Fold changes for discriminant metabolites among groups were estimated. Wilcoxon rank-sum tests were applied to determine the significance of the metabolites (*p* < 0.05) employing the online tool MetaboAnalyst. All the metabolites that significantly changed between groups regardless of the FC value were considered.

## Supporting Information Available

Details on the behavioral experiments and biochemical methodologies; information on qPCR primer design, ELISA characteristics, and metabolomics analysis; and additional metabolomics results (PDF)
